# A multimodal approach to acne mechanica associated to medical face masks using clinical examination, fluorescent photography, and in vivo reflectance confocal microscopy

**DOI:** 10.1007/s00403-025-04104-2

**Published:** 2025-03-17

**Authors:** Stefana Cretu, Denis Iorga, Mihai Dascalu, Carmen Maria Salavastru

**Affiliations:** 1 “Carol Davila” University of Medicine and Pharmacy, Bucharest, Romania; 2 Dermatology Research Unit, Colentina Clinical Hospital, Stefan cel Mare Street no 19-21, Bucharest, Romania; 3 Department of Computer Science, National University of Science and Technology Politehnica, Bucharest, Romania; 4 Academy of Romanian Scientists, Str. Ilfov, Nr. 3, Bucharest, 050044 Romania; 5 Paediatric Dermatology Department, Colentina Clinical Hospital, Stefan cel Mare Street no 19-21, Bucharest, Romania

**Keywords:** Acne, Acne mechanica, Multimodal, Reflectance confocal microscopy, Fluorescent photography, Medical face masks

## Abstract

With yet unclear pathogenesis, acne mechanica associated to medical face masks(MFM) gained increased attention during the COVID-19 pandemic. Between November 2021-January 2022 we conducted an exploratory, cross-sectional study evaluating the differences between the glabella and cheek-chin junction using clinical examination, fluorescent photography (FP), videodermatoscopy and in vivo reflectance confocal microscopy (RCM). Medical doctors from our country and 6th year medical students were invited. Participants self-identified as consistent MFM users during work hours. We analysed 19,432 images from 14 consecutive subjects, 11 females, 3 males, mean age,29.93 years (SD = 4.69). Clinical examination revealed more inflammatory lesions on the cheeks and chin (+ 9.35;95%CI + 2.31to + 16.39;*d* = 0.76, *P* =.01). FP considered ultraviolet red fluorescing spots(UVRFs) from the glabella and the cheek-chin junction, with significantly more spots in the former (+ 5.43;95%CI + 2.64to + 8.20; *d* = 1.12; *P* =.001). The average area in mm^2^ covered by the spots was significantly larger in the glabella (+ 3.26;95%CI + 1.43to + 5.08;*d* = 1.02; *P* =.002). RCM considered 9546 follicles, 4966 from the glabella, and 4580 from the cheek-chin junction. The cheek-chin junction had a significantly higher proportion of hyperkeratotic follicles than the glabella (+ 9%;95%CI + 2%to + 16%;*d* = 0.79;*P* =.01). In the dermal-epidermal junction, the cheek-chin region had a significantly higher proportion of follicles with signs of inflammation than the glabella (+ 10%;95% CI + 3%to + 18%; *d* = 0.80; *P* =.010). Perifollicular stratum corneum thickness was significantly lower in the cheek-chin junction, 19.56 μm (SD = 4.84) compared to the glabella, 24.25 μm (± 4.93) (-4 μm;95% CI-1 μm to– 8 μm; *r* =.88; *P* =.01). The distribution of UVRFs, known to correlate with microbial activity and sebum production, is similar to pre-pandemic studies, suggesting that in acne mechanica associated to MFM, follicular hyperkeratinisation, inflammation, and irritation, may outweigh other possible alterations.

**Supplementary Information:**

The online version contains supplementary material available at 10.1007/s00403-025-04104-2.

## Introduction

Acne mechanica caught increased attention during the initial stages of the COVID-19 pandemic, especially related to personal protective equipment(PPE), particularly medical face masks(MFM) or similar devices [2, 12, 22, 28, 53, 58]. The condition has been described before this time point; however, many aspects regarding its pathogenesis are yet to be uncovered [6, 16, 17, 51, 53].

In acne vulgaris, establishing the correct acne diagnosis and severity assessment are essential stages for optimal treatment [54]. Nevertheless, minimal diagnostic criteria for acne are currently unavailable [27] and there is little consensus regarding staging, with multiple possible approaches available [5].

Two of the most widely used methods in clinical practice for acne severity assessment are global grading, with several approaches available, or lesion counting [5], either during in-person visits or on photographs [46]. Attempts to use fluorescent photography(FP) for acne severity assessment have been described [41, 42, 45].

In vivo reflectance confocal microscopy(RCM) has been used for acne evaluation, with promising results and good correlation with clinical global grading [19, 21, 25, 35, 38].

Other non-invasive techniques have been explored for acne, both for lesion assessment and treatment monitoring [1, 10, 19–21, 30, 37, 38, 50]. Also, based on reflectance, optical coherence tomography (OCT) or dynamic optical coherence tomography may offer details regarding blood flow and inflammation, at deeper levels compared to RCM [30, 37, 38]. The level of detail of OCT is lower compared to RCM [1]. 

The aim of our study is to examine the differences between two facial areas, namely the cheek-chin junction (CCJunction) and glabella, in medical doctors (MD) and sixth year medical students (MS), in the context of MFM usage. We chose a multimodal approach and two distinct facial areas to evaluate potential differences and changes that the PPE might induce.

## Materials and methods

The study was approved by the Ethics Committee of “Carol Davila” University of Medicine and Pharmacy(18.10.2021/ No.28448) and was conducted according to the principles of the Declaration of Helsinki [3].Written informed consent was obtained from study participants.

Between November 2021-January 2022, we conducted an exploratory cross-sectional study involving 14 subjects, using clinical examination, FP, videodermatoscopy and RCM.

All participants self-identified as consistent users of MFMs during hours spent at work or throughout training.

**Fig. 1:**
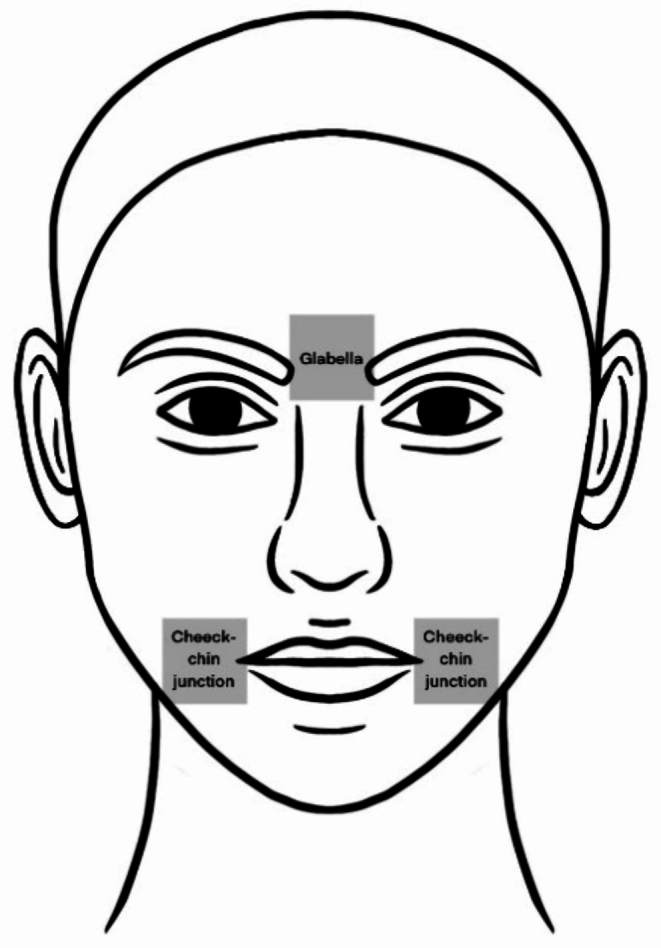
Schematic representation of examination sites, the boxes illustrate the facial regions examined

After clinical examination, photographs using Visia-CR (Canfield Scientific, Inc, USA) were acquired. Following, participants were examined using RCM in two areas of the face, the glabella, and the cheek-chin junction (Fig. 1). The glabella served as a control region, unexposed to the MFM, each participant served as their own control. A similar methodology was used in the study reported by Guenot et al. [25].

### Image analysis

Images were analysed using the ImageJ software version 2.9.0(National Institutes of Health, USA). We analysed a total of 98 images, comprising clinical photographs, photographs in ultraviolet A light (UVA), and videodermatoscopic images. Additionally, we examined a total of 56 8 × 8 mm image mosaics (VivaBlocks) and 98 stack images (VivaStaks).

### Clinical staging

Acne severity assessment was performed on the clinical photographs of the subjects, using the investigator’s global assessment (IGA) and manual lesion counting. Frontal and 45-degree side images acquired using cross polarized and parallel polarized light were analysed. We manually counted non-inflammatory and inflammatory acne lesions using the methodology of Lucky et al. [33]., considering each side of the forehead, each cheek, and the chin.

### Fluorescent photography

Frontal and 45-degree side images acquired in UVA using Visia-CR (Canfield Scientific, Inc, USA) were used to examine follicular red fluorescence by analysing ultraviolet red fluorescent spots (UVRFS). A custom 8 × 8 mm area from the glabellar and cheek-chin junction regions was selected. UVRFS were manually counted, their surface area was measured, and the percentage of area covered by UVRFS was calculated.

### Videodermatoscopy

After acquiring a standard 10 × 10 mm videodermatoscopic image using the VivaCam (Caliber ID Inc, USA) from each of the two regions considered. We selected an 8 × 8 mm section to enhance comparison with the fluorescent and RCM images. We manually counted follicles with yellow- brown plugs, corresponding to comedones [1, 31], measured their diameter, surface and calculated the percentage of covered area.

### In vivo reflectance confocal microscopy

Based on facial anatomy, we sampled similar areas of interest in all subjects, regardless of acne history, clinical presence of acne lesions. We used a standard image acquisition protocol [25, 35, 38] for the commercially available Vivascope 1500(Caliber ID Inc., USA), at 785 nm laser power, to acquire 8 × 8 mm VivaBloc mosaics from each of the two considered regions (i.e., glabella and cheek-chin junction).

Three image mosaics set 30 μm apart in depth were obtained. For each subject, a minimum of two VivaStacks were acquired for non-inflammatory acne lesions in each of the two areas investigated. Each image stack contained a standard sequence of 51 images, set apart by 3 μm, up to the papillary dermis, with a maximum average depth of 152 μm.

Hair follicles (HF) were manually counted and measured in diameter on image blocks from the epidermis (stratum granulosum-spinosum) and dermo-epidermal junction (DEJ) obtained from the glabella and cheek-chin junction.

The average depth for glabella samples was 43.66 μm, and for the CCJunction, 38.11 μm. We counted follicles with bright borders, corresponding to hyperkeratinisation [35, 38], content-filled follicles [19, 21, 25, 35, 38], follicles showing intra or perifollicular inflammatory signs [19, 21, 25, 35, 38], and follicles containing *Demodex folliculorum* [25].

Follicles showing grey amorphous material or granular grey debris [19] were considered content-filled [19, 35]. 

Signs of inflammation were considered the presence of at least one of the following features: “small bright dots” [35] inside or around HF, “white round-to-polygonal cells” [19] in the epidermis, “exocytosis” [35], and dermal inflammation [19, 25, 35, 38].

Consistent with the methodology described by Manfredini et al. [35, 38], we evaluated regular (infundibular diameter < 90 μm) and irregular follicles (infundibular diameter 90–200 μm and 200 μm) [38] to assess the “follicular density” [35, 38] and compared it between glabella and cheek-chin junction for each subject.

Stratum corneum (SC) thickness was assessed in the perifollicular area, not the interfollicular epithelium, because we aimed to evaluate HF features throughout all cutaneous layers.

It was measured using conventional methodology [39, 47], on image stacks for each subject in each facial region considered and computed as an average of at least two image stacks.

### Outcome measures and statistical analysis

Outcome measures, used to assess follicular features for the two areas investigated, were:, number, size and surface area percentage for UVRFS, number, size and percentage of area covered, for brown-yellow plugs, “follicular density” [35, 38], follicles with alterations, separately for each considered feature. We also analysed total number of acne lesions, number of inflammatory and non-inflammatory acne lesions.

Statistical analysis was performed using R 4.3.0, RStudio 2023.03.1 (Posit Software, PBC, USA).

First, we used descriptive statistics, counts and frequencies. We calculated means and standard deviations for the variables. Next, parametric, and non-parametric within-subjects 2-sided tests were performed.

The choice of parametric (Paired t-test) [29] or non-parametric (Wilcoxon paired signed-rank test) [56] procedure was made based on the results of the Shapiro-Wilk test. For each type of outcome measurement (e.g., total number of acne lesions), a Shapiro-Wilk test was applied to the difference between the glabella and cheek-chin measurements.

If the Shapiro-Wilk tests were not significant (*P* >.05) (i.e., showing that the differences are normally distributed), a parametric test was chosen; the corresponding effect sizes were also reported [11, 18].

A *P* <.05 was considered statistically significant for each conducted test. Exact *P* values were reported for all tests. Boxplot visualizations and confidence intervals were reported only for statistically significant differences. The Bonferroni correction was considered in interpreting the results presented in the discussion section.

## Results

During the 3-month study period, 14 consecutive subjects were enrolled, mean age, 29.93(SD = 4.69) years old, 11 females, 3 males; phototypes included were II-IV. There were 11 medical doctors (male: female ratio was 2:9) and 3 medical students (male: female ratio was 1:2).

### Image analysis and clinical staging

This study analysed 19,432 images in total. Table 1 presents the clinical assessment of acne severity using IGA in detail.

**Table 1:** General study population characteristics and clinical assessment of acne severity, the number (No.) of participants and percentages are reported, investigator global Assessment(IGA) was used for acne severity assessment

	Total number of participants *N* = 14
**No. (%) of participants with personal history positive for acne**	8(57%)
**No. (%) of participants with self-reported presence of facial acne lesions**	11(79%)
**No. (%) of participants with self-reported presence of topical anti acne-treatment**	4(29%)
**Treatment reported**	
No. (%) of participants using topical retinoids	1(25%)
No. (%) of participants using azelaic acid 10%	1(25%)
No. (%) of participants using fixed dose combination of benzoyl peroxide 4%, zinc sulphate 1%, retinol 0.5% and mandelic acid 1%,	1(25%)
No. (%) of participants using topical salicylic acid	1(25%)
No. (%) of participants with self-reported emollient use	12(86%)
**Clinical assessment according to the Investigator Global Assessment(IGA)**	
IGA 0	1(7%)
IGA 1	1(7%)
IGA 2	8(57%)
IGA 3	3(21%)
IGA 4	1(7%)

Total facial acne lesion count ranged from 59 to 327 lesions, with a mean of 156.71(SD = 71.63). For the entire face, the mean inflammatory lesion count was 34.21(SD = 20.06), while the mean non-inflammatory lesion count was 122.50(SD = 53.20).

**Fig. 2:**
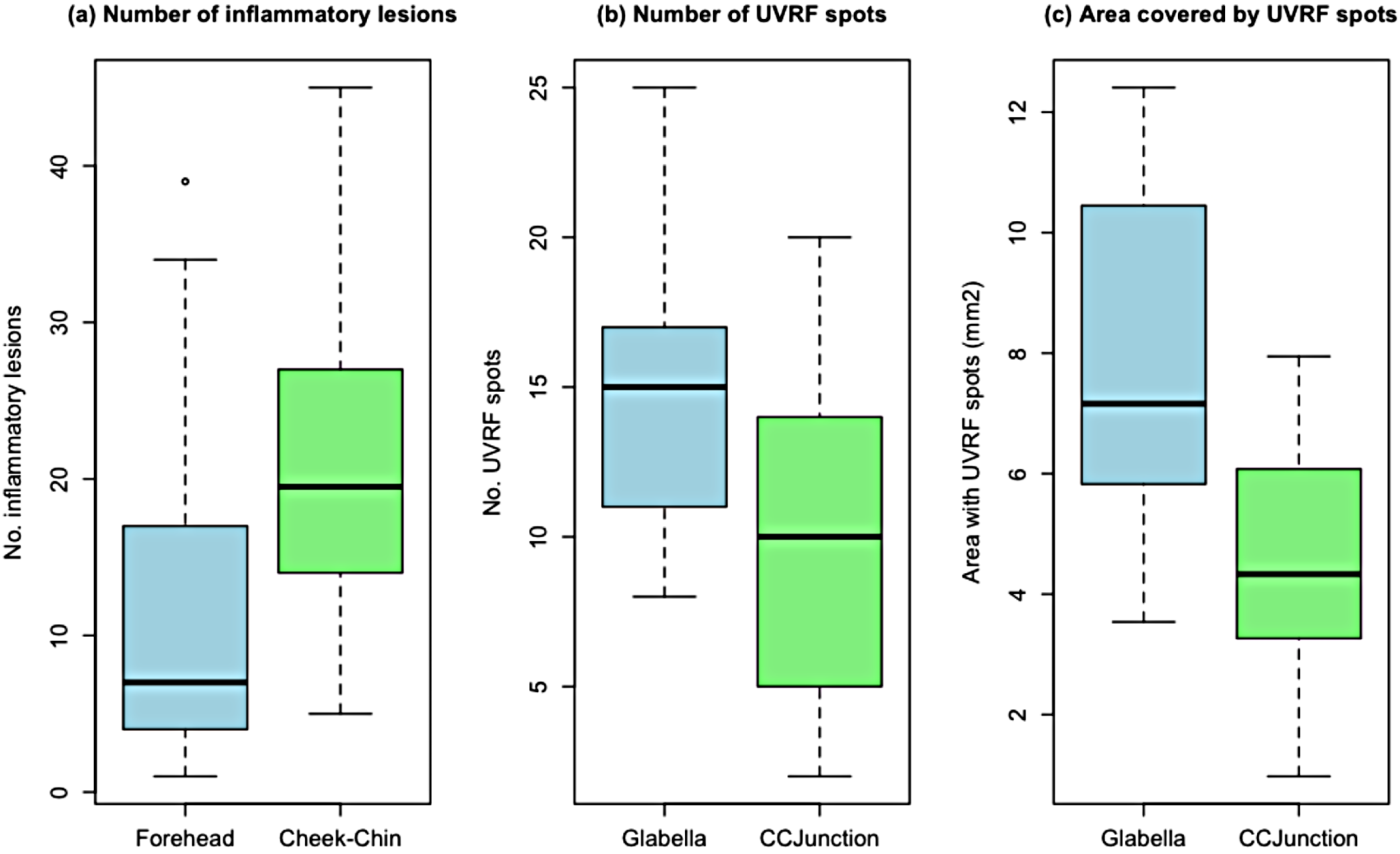
Significant differences between (**a**) inflammatory lesions from the forehead clinical region and both cheeks and chin, clinical regions and significant differences between glabella and cheek-chin junction (CCJunction), in terms of (**b**) number of ultraviolet red fluorescing spots, (**c**) area covered by the ultraviolet red fluorescing spots

The mean number of inflammatory lesions was 12.43(SD = 11.84) in the forehead and 21.78(SD = 11.63) on both cheeks and the chin. The difference between the two was statistically significant, with a medium effect size(+ 9.35;95%CI + 2.31to + 16.39;*d* = 0.76;*P* =.01). (See Fig. 2)

### Fluorescent photography

We analysed a total of 212 UVRFS from the glabella and 136 UVRFS from the CCJunction. On average, there were significantly more UVRFS in the glabella region with a large effect size (+ 5.43;95%CI + 2.64to + 8.20;*d* = 1.12;*P* =.001). Details are shown in Fig. 2; Table 2.

**Table 2:** Assessed features using clinical examination, fluorescent photography, and in vivo reflectance confocal microscopy (RCM), mean values, standard deviation (SD) are presented; characteristics of ultraviolet red fluorescing spots (UVRFS) are described; P values in italics are computed using non-parametric tests due to the non-normal distribution of differences

	Forehead (clinical view) Mean(SD)	Cheek and chin areas (clinical view) Mean(SD)	*P*.value
**Acne lesion count**			
Inflammatory acne lesions	12.43 (± 11.84)	21.78(± 11.63)	0.013
Non-inflammatory acne lesions	61.29(± 41.62)	61.21 (± 16.59)	0.993
Open comedones	50.21 (± 37.39)	48.64 (± 13.41)	*0.818*
Closed comedones	11.07 (± 8.26)	12.57 (± 7.80)	0.559
**Ultraviolet red fluorescing spots (UVRFS)**	**Glabella** **Mean(SD)**	**Cheek-chin junction** **Mean(SD)**	**P.value**
Number of UVRFS	15.14(*±* 4.68)	9.71(*±* 5.39)	0.001
Area covered by UVRFS	7.88(± 2.73) mm^2^	4.62(± 2.14) mm^2^	0.002
Diameter of the UVRFS	865.91(± 181.84) µm.	839.50(± 187.74) µm	0.637
**Dermatoscopy**			
Number of follicles with brown-yellow plugs	80.43(*±* 20.43)	87.93(*±* 44.51)	*0.747*
Area covered by follicles with brown-yellow plugs	6.83(*±* 1.88) mm^2^	5.75(*±* 2.67) mm^2^	0.148
In vivo **reflectance confocal microscopy(RCM)**			
Infundibulum size	119.62(± 21.09) µm	129.59(± 29.51) µm	0.126
Total number of follicles	354.67(± 65.82)	327.17(± 74.20)	0.231
Normal follicles (< 90 μm)	151.50(± 71.24)	148.92(± 76.44)	0.902
Proportion of normal follicles (< 90 μm)	0.42(± 0.15)	0.44(± 0.18)	0.639
Medium follicles (90–200 μm)	168.96(± 51.54)	136.17(± 54.72)	0.108
Proportion of medium follicles (90–200 μm)	0.48(± 0.12)	0.42(± 0.15)	0.238
Large follicles (> 200 μm)	34.21(± 20.48)	41.86(± 18.57)	0.210
Proportion of large follicles (> 200 μm)	0.10((± 0.06)	0.13(± 0.06)	0.103
Bright border follicles	271.32(± 71.38)	279.78(± 63.85)	0.674
Proportion of bright follicles	0.76(± 0.14)	0.86(± 0.09)	0.010
Content-filled follicles	158.42 (± 43.43)	157.46(± 60.05)	0.931
Proportion of content-filled follicles	0.44(± 0.11)	0.47(± 0.14)	0.265
Follicles with inflammation	188.57(± 93.29)	208.78(± 72.78)	0.207
Proportion of follicles with inflammation	0.54(± 0.27)	0.65(± 0.21)	0.010
Follicles with Demodex folliculorum	7.25(± 9.61)	14.10(± 20.57)	*0.533*
Proportion of follicles Demodex folliculorum	0.02(± 0.03)	0.04(± 0.07)	*0.408*
Perifollicular SC thickness	24.25(± 4.93)	19.56(± 4.84)	*0.012*

The overall average percentage of area covered by the UVRFS was 7.88 mm^2^(12.31%) for the glabella sections and 4.62 mm^2^(7.21%) for the CCJunction sections.

The average area covered by UVRFS was significantly larger in the glabella sections than in the CCJunction sections, with a large effect size (+ 3.26;95%CI + 1.43to + 5.08; *d* = 1.02;*P* =.002).

The mean diameter of the UVRFS was larger in the glabella than in the CCJunction. Nonetheless, the difference between the two areas in terms of diameter was not statistically significant (Table 2).

### Videodermatoscopy

In total, we found 1,126 comedones in the glabella and 1,231 in the CCJunction. The differences regarding the mean number of comedones, the mean area and the mean percentage of area covered were not statistically significant between the two regions (Table 2**).**

### In vivo reflectance confocal microscopy

For this study, we considered a total number of 9,546 follicles, 4,966 follicles from the glabella region and 4580 from the CCJunction region. The number of follicles was here computed as the average of measurements between the intraepidermal and dermal-epidermal junction depths. There was no significant difference between the average number of follicles in the glabella and CCJunction regions.

Data regarding each individual depth separately are available in Online Resource 1.

### Bright-border follicles

The mean number of bright-border (hyperkeratotic) follicles for the two measurement depths considered are detailed in Table 2.

At the epidermal level, there were 3798 hyperkeratotic follicles in the glabella and 3971 in the cheek-chin region. A statistically significant difference could be observed between the glabella and the CCJunction regions as regards the proportion of bight border follicles out of total follicles, with a medium effect size(+ 9%;95%CI + 2%to + 16%;*d* = 0.79;*P* =.01).

### Follicles with inflammation

For each of the two measurement depths considered, the mean number of follicles showing intra-or peri-follicular signs of inflammation is shown in Table 2.

The proportion of follicles showing intra-or peri-follicular signs of inflammation was higher in the CCJunction than in the glabella. The difference was statistically significant with a large effect size (+ 10%;95% CI + 3%to + 18%; *d* = 0.80; *P* =.010).

### Perifollicular SC thickness

The mean number of samples was 3.5 (SD = 1.22) in the glabella region, and 3.57 (SD = 1.08) in the CCJunction region. There were no statistically significant differences between the number of slices taken from the glabella and cheek-chin regions.

**Fig. 3:**
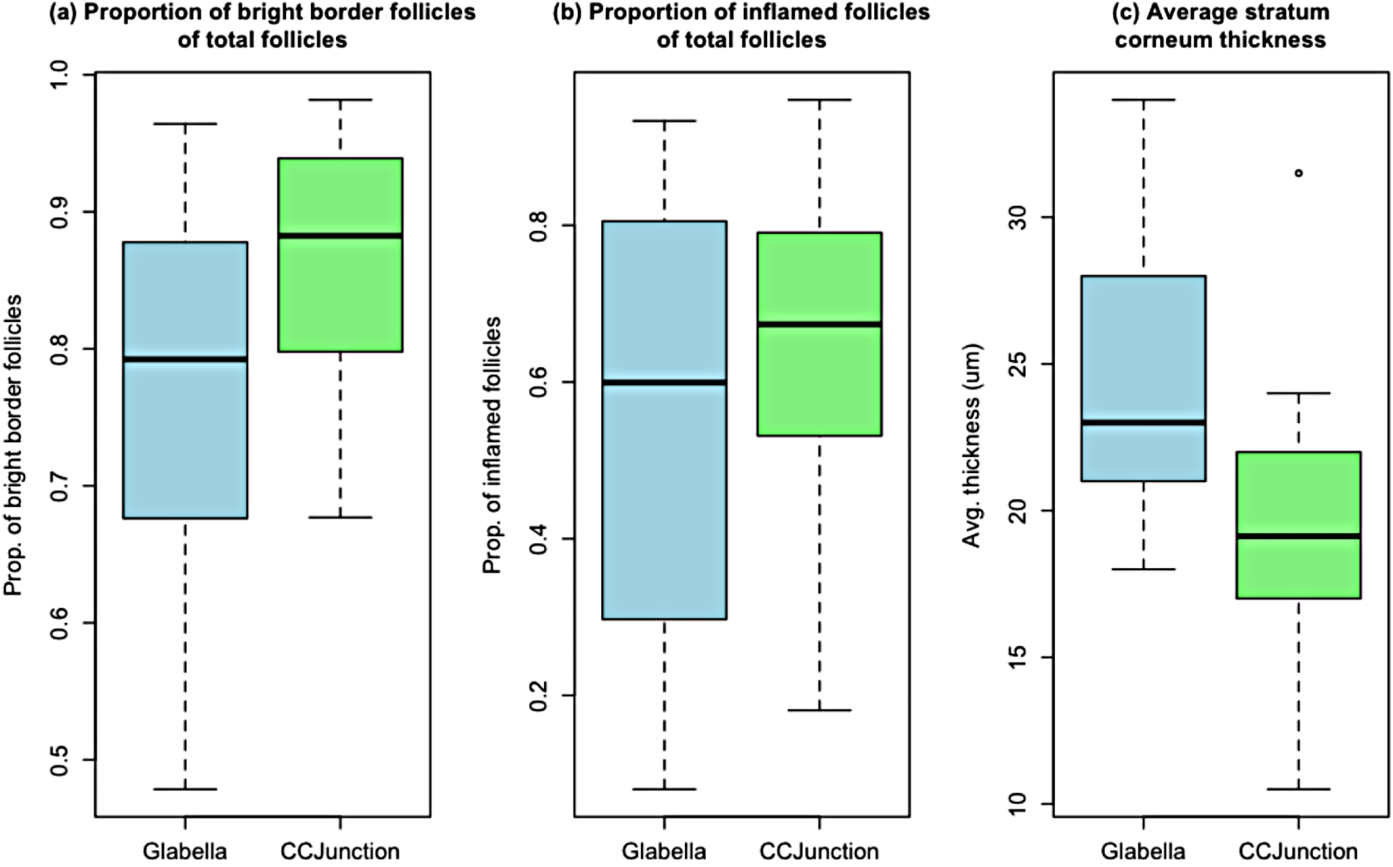
Significant differences between glabella and CCJunction, in terms of (**a**) proportion of bright border follicles at the epidermal level, (**b**) proportion of inflamed follicles at the dermal − epidermal junction level, (**c**) average stratum corneum thickness

The mean SC thickness was 24.25 μm (SD = 4.93) in the glabella, compared with 19.56 μm (SD = 4.84) in the CCJunction region. The differences were statistically significant, with a large effect size (-4 μm;95% CI-1 μm to– 8 μm; *r* =.88; *P* =.01). Data is detailed in Table 2; Fig. 3.

## Discussion

Multiple observations and hypotheses regarding the pathogenesis of acne mechanica have been reported [16, 52, 53, 58]. During the COVID-19 pandemic, although many reports were presented regarding acne lesions associated with PPE, the majority were self-reported by participants because of limitations regarding interpersonal contact [12, 13, 26, 43, 52].

The study explored differences in measurements between face regions covered and not covered by MFM. It must be noted that only differences in UVRF spots and area were significant when considering the Bonferroni correction. Nonetheless, due to the exploratory nature of the research, all significant P values were viewed as signals for potential hypotheses. As such, these were interpreted in the light of existing research.

In this study, the clinical assessment of subjects found the presence of open and closed comedones in the area covered by the MFM, confirming that subjects were affected by acne. Moreover, we found significantly more inflammatory lesions in the area covered by the MFM.

Clinical evaluation of acne severity may be subject to increased intra- and interrater variability; for this reason, additional staging techniques have been described [5, 15, 19, 44, 45]. The current report used additional tools to enrich the findings provided by clinical examination alone.

Analysing photographs acquired using UVA light, we found that the number of UVRFs and the average area they covered were higher in the glabella compared to the cheek-chin junction.

Similarly, Dobrev reported in 2010 that the forehead, nose and chin have more UVRFs, covering a larger surface than the cheeks [15]. In their 2011 report, Choi et al. reported that the fluorescence pattern was more abundant in the T-zone, comprising the nose, forehead, and chin, and that it tended to descend with increasing age. They noted that in the U-zone, comprising the cheeks, with increasing age, fluorescence tended to concentrate towards the facial centre [9]. Also, in 2016 Baek et al. reported the highest number of porphyrin spots on the forehead [4].

Ultraviolet red fluorescence has been shown to be the result of microbial activity within the sebaceous follicle [45, 49, 57]. Sebum lacking microbial activity does not show fluorescence [49, 57].

UVRFs are known to correlate with seborrhoea [9, 15]. Our observations regarding the UVRFs are aligned with these pre-pandemic studies [4, 9, 15], suggesting that microbiota alterations and increased sebum production associated with MFM usage may not be the main factors involved in the pathogenesis of this subtype of mechanical acne.

Moreover, Wongtada et al. [55]. found that the microbiota in the area exposed to the MFM was similar to unexposed sites in subjects affected by mild acne. Our study, in addition to mild acne, also included subjects with moderate or severe acne, suggesting that the results may be applicable to other degrees of acne severity.

The use of dermatoscopy for acne has been reported [1, 31]. RCM is a detailed technique also useful for acne evaluation; however, in contrast to dermatoscopy, it may prove time-consuming [1, 14]. Considering these aspects, we considered dermatoscopic images to identify parameters that may be correlated to confocal ones for a more rapid evaluation. In our setting for mechanical acne, this could not be achieved, suggesting that the detailed results RCM provides are challenging to replace.

Pre-pandemic reports, showed that hyperkeratinisation is the main event in acne [19, 25, 35, 38]. Our finding, that bright border follicles are more numerous in the cheek-chin region compared to the glabella in the epidermal layer, suggest that hyperkeratinisation is an important event also in mechanical acne.

Guenot et al. found that in adult females with acne, the subtype with diffuse lesions presents with morphologic changes involving follicles from both the forehead and the mandible, whereas the acne subtype with predominant mandibular involvement only shows changes in the mandibular area, with the forehand uninvolved [25]. Our study had a significant female predominance. Nevertheless, females affected by acne presented with diffuse clinical lesions. In addition to adult females affected by acne, we also included males, with findings consistent with acne changes in the area exposed to the MFM.

Moreover, our report supports previous findings that bright border follicles are an important parameter for acne evaluation [19, 21, 25, 38], which may be used in the future as a minimal diagnostic criterion.

We found significantly more HF with signs of inflammation in the cheek-chin region compared to the glabella at the DEJ level. Coupled with the observation regarding the increase in bright border follicles in the epidermal layer, this finding suggests acne associated inflammation in the area covered by the MFM.

Both at the epidermal level, as well as at the DEJ the proportion of mite positive follicles was higher in the cheek-chin region than in the glabella; however, we identified more mites in the DEJ than in the upper layers, possibly because Demodex mites were more visible as we examined deeper in the HF.

Using two section planes at distinct depths enabled us to find all these features. When performing the analysis on a single section (for example, at the epidermal level), the findings considering inflammation may have been missed. Similarly, those considering hyperkeratinisation would have been missed if we would have only performed the examination in the dermal-epidermal junction level.

SC was thinner in the cheek-chin area compared to the glabella, supporting the skin barrier damage the MFM may induce [52]. SC damage pleads for an irritant, not an allergic mechanism [24]. No spongiosis was noted, suggesting that allergy [24] from mask components is less likely. The SC thickness was greater in our study for both measurement sites than in previous reports [7, 47]. One reason for the difference between the two in terms of SC thickness could reside in our analysis of perifollicular SC. Another reason could be caused by between-subjects variations as well as variations within a particular site, also reported by Robertson and Rees [47]. Although artefacts owed to skin furrows may be possible [7, 23], having applied the same method between glabella and cheek-chin analysis reduces bias regarding measurement site choice, in our sample. Also, having taken multiple samples from each considered site enabled us to compute the average measurement, thus reducing possible variability.

Most of the subjects included, 12(86%) reported using emollients; as such, the difference may have been even greater in their absence.

Strengths of this study include analysing acne mechanica in health care providers and medical students in a real-world setting and comparing two areas of the face using the same measurement area. Also, another strength is the use of fluorescent photography, with UVRFS assessment, known to correlate with seborrhoea and microbial activity [15, 57] coupled with RCM, known to be useful in assessing HF alterations and skin barrier damage [10, 19, 25, 35, 38].Finally, analysing two distinct measuring depths, using RCM, on 8 × 8 mm mosaics, provided increased samples and follicles to enable relevant observations.

This study adds to the existing knowledge an evaluation through objective, non-invasive, measurements, hypothesising that in acne mechanica the interplay between irritation, hyperkeratinisation, and inflammation outweighs other possible, theoretic, alterations.

The limitations of our study include the lack of a control group with people unaffected by acne and not exposed to the MFM. Also, due to the observational design of the study, proving causality is challenging. The sample size is another limitation. Addressability was low since in-person visits require more effort and time from participants. Previous studies focusing on RCM use in acne are also limited in their sample size [8, 19, 21, 25, 32, 34–36, 38, 40, 48]. One reason may be that investigating the samples taken from the subjects in great detail limits the possibility of evaluating a large cohort.

Another study limitation consists of having chosen the glabella and not a distinct part of the forehead as a control region. The glabella may contain eyebrow hairs, which may be terminal or intermediate, possibly influencing the follicular diameter. Nevertheless, variations in follicle type may also be present on the cheek or chin, for example, the beard in males. We had a within-subjects comparison and analysis. It is expected that such variations are consistent within the same person. Also, some of the subjects included were using some type of anti-acne medication.

To conclude, our observations using fluorescent photography are aligned with pre-pandemic findings regarding the distribution of UVRFS. The results from our study suggest that the pathogenesis of MFM associated acne mechanica, follicular hyperkeratinisation, inflammation, and irritation outweigh possible alterations in follicular microbiota and excess sebum production. Our observations may guide future confirmatory studies focused on treatment for acne mechanica and the education of patients, especially in avoiding further irritation from unsuitable treatments and unnecessary exposure to antibiotics.

## Electronic supplementary material

Below is the link to the electronic supplementary material.

Supplementary Material 1

## Data Availability

The data supporting this study’s findings is available upon reasonable request from the corresponding author.
