# Supplementary material for: A multimodal approach to acne mechanica associated to medical face masks using clinical examination, fluorescent photography, and in vivo reflectance confocal microscopy
Source: Arch Dermatol Res. 2025 Mar 17;317(1):578. doi: 10.1007/s00403-025-04104-2 (PMC11913915; doi:10.1007/s00403-025-04104-2)
Supplement: Supplementary file 1 — Supplementary Material 1 [file 403_2025_4104_MOESM1_ESM.pdf]

Supplementary Information referring to the article:

A multimodal approach to acne mechanica associated to medical face masks using clinical examination, fluorescent photography, and in vivo reflectance confocal microscopy

Archives of Dermatological Research

Stefana Cretu[1,2](ORCID number 0000-0002-1127-5726) Denis Iorga[3], Mihai Dascalu[3,4] (ORCID number 0000-0002-4815-9227), Carmen Maria Salavastru[1,5]

[1] “Carol Davila” University of Medicine and Pharmacy, Bucharest, Romania

[2] Dermatology Research Unit, Colentina Clinical Hospital, Bucharest, Romania,

[3] Department of Computer Science, National University of Science and Technology POLITEHNICA Bucharest, Romania,

[4] Academy of Romanian Scientists, Str. Ilfov, Nr. 3, 050044 Bucharest, Romania,

[5] Paediatric Dermatology Department, Colentina Clinical Hospital, Bucharest, Romania,

Corresponding author:

Dr. Stefana Cretu; „Carol Davila” University of Medicine and Pharmacy, Dermatology PhD Student, Dermatology Research Unit, Colentina Clinical Hospital, Stefan cel Mare Street no 19-21, Bucharest, Romania, +4 021 317.32.45, stefana_spiridon@yahoo.com

Online Resource 1 : Assessed features, specifically for each individual depth, using *in vivo* reflectance confocal microscopy (RCM), mean values, standard deviation (SD) are presented; p.values in italics are computed using non-parametric tests due to the non-normal distribution of differences.

	**Glabella Mean(SD)**	**Cheek-chin junction Mean(SD)**	**p.value**	**Glabella (proportion of total)**	**Cheek-chin junction (proportion of total)**	**p.value**
**Total number of follicles**						
Epidermal level	360.57(±85.78)	339.93(±81.79)	0.500	-	-	-
Dermal-epidermal junction	348.78(±62.74)	314.42(±71.49)	0.073	-	-	-
**Normal follicles (<90µm)**						
Epidermal level	186.00(+93.17)	174.00(+86.01)	0.684	2604 (0.51)	2436 (0.51)	0.787
Dermal-epidermal junction	117.00(±60.01)	123.85(±87.92)	0.779	1638 (0.33)	1734 (0.39)	0.462
**Follicles with infundibulum diameter between 90-200 µm**						
Epidermal level	139.71(+54.04)	117.57(+47.27)	0.219	1956 (0.38)	1646 (0.34)	0.337
Dermal-epidermal junction	198.21(±58.50)	154.78(±71.77)	0.095	2775 (0.56)	2167 (0.49)	0.313
**Large follicles (>200µm)**						
Epidermal level	34.85(+23.27)	48.35(+26.21)	0.128	488 (0.09)	677 (0.14)	0.129
Dermal-epidermal junction	33.57(±21.78)	35.35(±18.91)	0.786	470 (0.09)	495 (0.11)	0.401
**Bright border follicles**						
Epidermal level	280.64(±87.74)	299.28(±72.65)	0.554	3929 (0.77)	4190 (0.88)	0.028
Dermal-epidermal junction	262.00 (±70.77)	260.28(±72.04)	0.921	3668 (0.75)	3644 (0.82)	0.109
**Content-filled follicles**						
Epidermal level	165.50(±55.65)	164.00(±66.47)	0.934	2317 (0.45)	2296 (0.48)	0.662
Dermal-epidermal junction	151.35(±40.53)	150.92(±59.69)	0.967	2119 (0.43)	2113 (0.48)	0.216
**Follicles with inflammation**						
Epidermal level	186.85(±92.14)	211.00(±80.41)	*0.696*	2616 (0.51)	2954 (0.62)	0.058
Dermal-epidermal junction	190.28(±99.59)	206.57(±67.15)	0.315	2664 (0.54)	2892 (0.65)	0.004
**Follicles with Demodex folliculorum**						
Epidermal level	6.64(±9.81)	12.14(±15.36)	*0.319*	93 (0.01)	170 (0.03)	*0.214*
Dermal-epidermal junction	7.85(±10.12)	16.07(±29.67)	*0.611*	110 (0.02)	225 (0.05)	*0.447*
